# Sustained Ventricular Tachycardia In An Apparently Healthy Heart: A Very Localized Left Dominant Arrhythmogenic Cardiomyopathy

**DOI:** 10.1016/s0972-6292(16)30819-1

**Published:** 2014-12-15

**Authors:** Alberto Cresti, Francesco De Sensi, Silva Severi, Gennaro Miracapillo

**Affiliations:** Cardiology Department, Misericordia Hospital, Grosseto, Italy

**Keywords:** Arrhythmogenic Cardiomyopathy, Ventricular Tachycardia, Cardiac Magnetic Resonance

## Abstract

A 62-year-old man admitted for presyncope presented two symptomatic sustained ventricular tachycardia with right bundle branch morphology and inferior axis suggesting a pathology of the left ventricular lateral wall, the site where Cardiac Magnetic Resonance demonstrated a thinned, hypokinetic segment with fibro-fatty subepicardial infiltration. A very localized Left Dominant Arrhythmogenic Cardiomyopathy was diagnosed and an ICD implanted.

## Case Report

**Figure 1:**
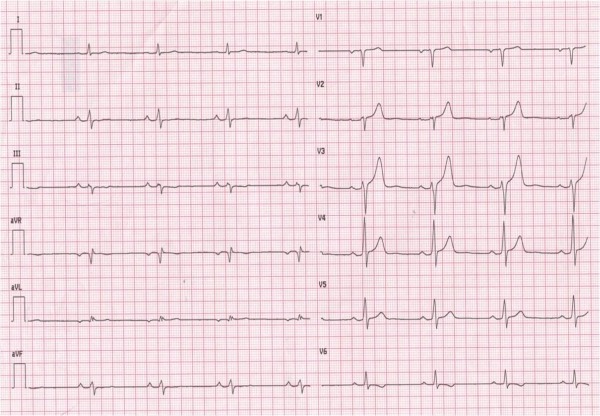
Baseline electrocardiogram (see text for description).

A 62-year-old man was admitted to our Cardiology Department for chest pain, presyncope and mild elevation of cardiac enzymes (troponin= 1.75 ng/ml). A fast echocardiogram in the ER didn't show significant abnormalities. Electrocardiogram (ECG) revealed low QRS voltage in limb leads, especially in aVL with a notch, no progression of R wave in V5-V6,T-wave inversion in V6 and T-wave flattening in inferior leads (Figure 1). Coronary angiography was normal.

**Figure 2:**
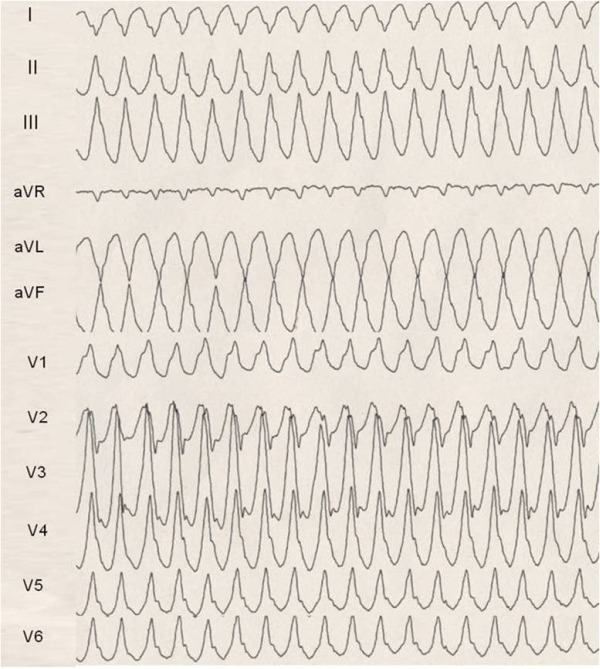
Ventricular Tachycardia induced at EP study. RBBB morphology and inferior axis with negative QRS axis in aVL and I leads suggest a left ventricle-lateral wall origin. Dominant R waves in V3-V6 leads point at a basal exit of the tachycardia, close to the mitral annulus. Furthermore one can notice slurring of QRS with long QRS duration (180 msec), QS pattern in I and aVL with a late notch, pseudo-delta wave in V1>34 msec: all these elements suggest an epicardial origin of the VT.

During the first 48 hours the in-hospital ECG continuous monitoring showed frequent isolated ventricular ectopic beats and an Electrophysiological Study was proposed to the patient who accepted. With a simple stimulation protocol from the RV apex (drive -S1-S2 = 600-280-270 msec) a fast ventricular tachycardia was induced. The VT had a right bundle branch (RBB) morphology and inferior axis, with dominant R wave in all precordial leads (Figure 2). It was poorly tolerated and terminated by overdrive pacing.

2D-echocardiography repeated by a skilled operator revealed a mild enlargement of the left ventricle (End Diastolic Diameter: 60 mm, End Diastolic Volume: 80ml/mq), mid and basal infero-lateral hypokinesia with preserved global function, EF: 58%. Right ventricle was normal. Cardiac MRI was planned.

**Figure 3:**
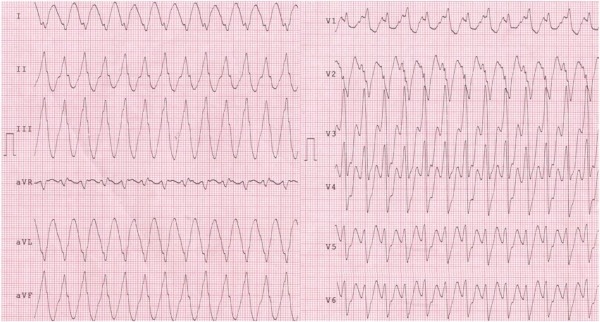
Clinical Ventricular Tachycardia recorded during the hospitalization. As long as the previous induced VT, RBBB morphology and inferior axis with QS complex in aVL and I leads indicate a left ventricle-lateral wall origin. Differently, an RS complex in V3-V6 leads point at a more medium-apical exit of the tachycardia. Moreover there are few elements wich suggest an epicardial origin of the VT (notice, among others, such a precordial pattern break in V1-V2).

The night before patient presented chest pain and sweating while ECG monitoring documented a sustained ventricular tachycardia with right bundle branch (RBB) morphology and inferior axis, xylocaine sensitive (Figure 3). This VT was similar but not identical to the previous induced one, presenting an RS complex in V3-V6.

The VTs morphology suggested a reentry circuit in the LV lateral wall around the localized fibrous-fatty infiltration strip with two different exit sites: a basal site, close to the mitral annulus for the EP induced VT, and more apical for the clinical VT . Both VTs seemed to respect most of criteria previously described to suspect an epicardial origin [1].

**Figure 4:**
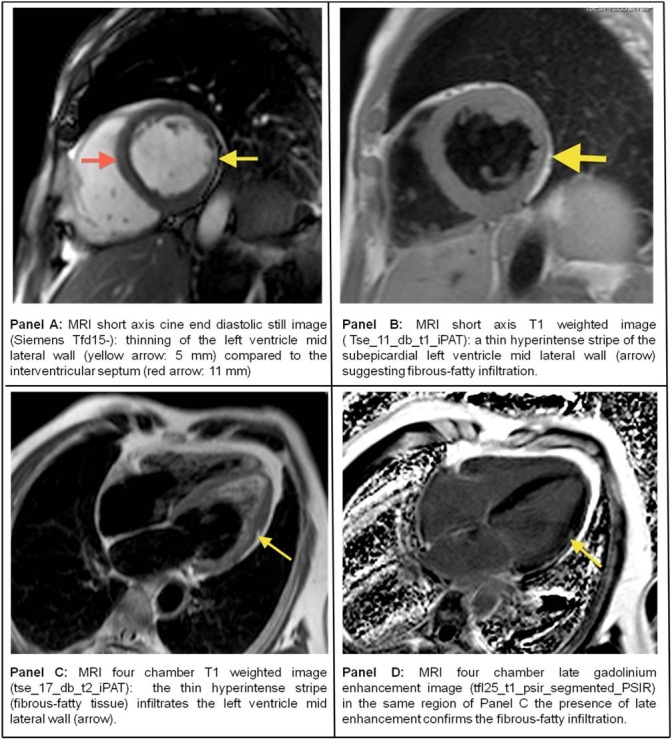
Cardiac MRI images showing morphological alterations in the left ventricle lateral wall (see Panels for details).

Successively Cardiac Magnetic Resonance (CMR) showed thinning of the lateral wall (Figure 4, Panel A) with fibrous-fatty subepicardial infiltration (T1W in Figure 4 - Panel B and C , and LGE sequences in Figure 4 - Panel D) typical of a left-dominant arrhythmogenic cardiomyopathy (LDACM). No sign of fibrous-fatty replacement in the right ventricle was present.

Antiarrhythmic therapy with amiodarone and metoprolol was started and a dual chamber ICD was implanted. The first month ICD follow up visit revealed one silent VT episode cardioverted by first ATP and 8 months later a VT symptomatic for syncope cardioverted by DC shock. Ablation therapy was proposed to the patient who refused. Successively patient developed amiodarone induced thyrotoxicosis necessitating interruption of the drug and replacement with sotalol.

LDACM is an under-recognized disorder that should be suspected in patients with unexplained left ventricular arrhythmias exceeding the degree of cardiac pathology/dysfunction. Clinical features include infero-lateral T waves inversion, ectopic ventricular beats or VT with RBB morphology, a thinned and/or hypokinetic LV segments and signs of fibrous-fatty replacements on histology or LGE imaging on CMR [2]. Diagnostic accuracy of non invasive detection of myocardial fibrosis in arrhythmogenic cardiomyopathy using delayed-enhancement magnetic resonance imaging is very high (specificity and positive predictive value close to 100%) [3].

Our case is challenging because of the initial differential diagnosis with acute coronary syndrome necessitating coronary angiography. Then myocarditis could be excluded by T1w and LGE CMR sequences. It's also interesting because it fulfils each clinical features described by group of McKenna and shows how electrical phenomena are related to the structural alterations visualized on the cardiac imaging.

To our knowledge, it's the first described case of LDAC localized only to a single wall of the left ventricle, without involving the inter-ventricular septum [4]. Thinning of the lateral wall and the epi-myocardial distribution of the fibrous-fatty strip makes an endomyocardial biopsy dangerous and of limited value, due to it's poor sensitivity, even if performed under CARTO guidance as previously described [5]. However, in presence of all other clinical features, EMB is not necessary for the diagnosis.
